# Epigenetic regulation and factors that influence the effect of iPSCs-derived neural stem/progenitor cells (NS/PCs) in the treatment of spinal cord injury

**DOI:** 10.1186/s13148-024-01639-5

**Published:** 2024-02-21

**Authors:** Yubiao Yang, Boyuan Ma, Jinyu Chen, Derong Liu, Jun Ma, Bo Li, Jian Hao, Xianhu Zhou

**Affiliations:** 1 The Second Affiliated Hospital, Guangzhou Medical University, Guangzhou, 510260 People’s Republic of China; 2 Department of Orthopedics, Tianjin Medical University General Hospital, Tianjin, People’s Republic of China; 3 Department of Orthopedics, Beijing Luhe Hospital, Capital Medical University, Beijing, People’s Republic of China

## Abstract

Spinal cord injury (SCI) is a severe neurological disorder that causes neurological impairment and disability. Neural stem/progenitor cells (NS/PCs) derived from induced pluripotent stem cells (iPSCs) represent a promising cell therapy strategy for spinal cord regeneration and repair. However, iPSC-derived NS/PCs face many challenges and issues in SCI therapy; one of the most significant challenges is epigenetic regulation and that factors that influence this mechanism. Epigenetics refers to the regulation of gene expression and function by DNA methylation, histone modification, and chromatin structure without changing the DNA sequence. Previous research has shown that epigenetics plays a crucial role in the generation, differentiation, and transplantation of iPSCs, and can influence the quality, safety, and outcome of transplanted cells. In this study, we review the effects of epigenetic regulation and various influencing factors on the role of iPSC-derived NS/PCs in SCI therapy at multiple levels, including epigenetic reprogramming, regulation, and the adaptation of iPSCs during generation, differentiation, and transplantation, as well as the impact of other therapeutic tools (e.g., drugs, electrical stimulation, and scaffolds) on the epigenetic status of transplanted cells. We summarize our main findings and insights in this field and identify future challenges and directions that need to be addressed and explored.

## Methods

In this research, we performed a systematic literature review to identify existing literature related to our topic from multiple databases by using a clear search strategy and specific inclusion and exclusion criteria. Then, we conducted quality assessment, data extraction, comprehensive analysis and discussion.

We screened four different databases: PubMed, Web of Science, Scopus and China National Knowledge Infrastructure (CNKI). Our search period was from the 1st of January 2000 to the 31st of March 2023. Our search keywords were as follows: (iPSCs OR induced pluripotent stem cells) AND (NS/PCs OR neural stem/progenitor cells) AND (SCI OR spinal cord injury) AND (epigenetics OR DNA methylation OR histone modification OR chromatin structure). Our logical operators were AND and OR. Our filtering conditions were as follows: English or Chinese language; the document type was an original article or review article; and the document subject was biomedical. A typical example search was as follows: (iPSCs OR induced pluripotent stem cells) AND (NS/PCs OR neural stem/progenitor cells) AND (SCI OR spinal cord injury) AND (epigenetics OR DNA methylation OR histone modification OR chromatin structure) AND (language: English OR language: Chinese) AND (document type: original article OR document type: review article) AND (subject area: biomedical). We identified a total of 1234 documents, of which 1056 documents met our filtering conditions; 178 documents did not meet our filtering conditions, mainly because due to language conditions, document type or document subject.

We included documents involving epigenetic aspects of iPSCs-derived NS/PCs in spinal cord injury treatment and excluded duplicated documents and documents that were of low quality or were irrelevant to our topic or lacking key information. First, we excluded 456 documents by reading the title and abstract of the documents; these documents were mainly excluded because the documents were irrelevant to our topic or lacked key information. Then, we excluded 312 documents by reading the full text of the documents; these documents were mainly excluded because the documents were duplicated, were of low quality, or were irrelevant to our topic. Finally, we included 288 documents as the data source for our literature review.

Our quality assessment and data extraction methods were as follows. We used AMSTAR 2 (A Measurement Tool to Assess systematic Reviews) as our assessment tool, and scored the quality of each document into three levels: high quality, medium quality and low quality. We extracted the following data types and variables: basic information of the document (such as author, title, publication year, and publication journal), the research purpose, the method, results, conclusion, limitations and prospects. We organized these data into a data table, and performed descriptive statistics and thematic analysis.

We conducted systematic, comprehensive and innovative analysis and discussed the mechanisms, effects and safety of iPSC-derived NS/PCs in the treatment of spinal cord injury from the perspective of epigenetics. We summarized the epigenetic characteristics and influencing factors for iPSC-derived NS/PCs in the treatment of spinal cord injury, discussed the epigenetic advantages and challenges of iPSC-derived NS/PCs in the treatment of spinal cord injury, and proposed epigenetic optimization strategies and future directions for the use of iPSC-derived NS/PCs in the treatment of spinal cord injury.

## Introduction

Spinal cord injury (SCI) is a devastating neurological disorder that leads to necrosis or apoptosis in the neurons and glial cells of the spinal cord at the site of injury, thus resulting in the loss of neural tissue and the disruption of neural circuits. It is estimated that approximately 2.7 million people had experienced SCI worldwide, with an annual incidence of approximately 180,000 cases. The pathophysiology of SCI is characterized by both primary injury and secondary injury. Primary SCI refers to the immediate damage inflicted on spinal cord tissues by mechanical trauma. In contrast, secondary SCI encompasses a cascade of intricate pathological events that unfold following the primary injury. This sequence includes inflammation, ischemia, edema, necrosis, apoptosis, demyelination, and the formation of glial scars. These processes cumulatively exacerbate spinal cord damage and contribute to further functional loss. This causes partial or complete impairment of sensory and motor functions and also generates a significant burden for both patients and society [1–5]. Currently, effective treatments for SCI are very limited, relying mainly on surgery, drugs and rehabilitation to alleviate symptoms and complications; however, these treatments fail to achieve substantial recovery of neurological function [6]. Therefore, developing novel therapeutic approaches that can promote spinal cord regeneration and repair is an important goal of SCI research.

Neural stem cell (NSC) transplantation is a promising therapeutic strategy that can be used to improve neurological function in SCI patients by promoting neuronal and axonal regeneration, inhibiting inflammation and scar formation, and providing neurotrophic factors [7]. Nevertheless, the application of NSC transplantation for the treatment of SCI is hindered by several critical challenges. These include the limited availability of NSC sources, the low survival and differentiation efficiency of the transplanted cells, and their inadequate compatibility and integration with the host tissues. Therefore, identifying a safe, efficient and controllable source of NSCs, as well as optimizing the phenotype and functionality of transplanted cells in vivo, is critical if we are to improve the efficacy of NSC transplantation for the treatment of SCI. [8]

Over recent years, cell therapy has become a hot topic and a new frontier in the field of SCI therapy. The basic principle of cell therapy is to transplant regenerative cells to the injury site to replace damaged neural tissue, reconstruct neural circuits, provide nutritional support, and inhibit inflammatory responses and scar formation [6, 9].

Several types of cells have been used for cell transplantation therapy in SCI However, the choice of cell type for transplantation is critical; this is because different cell types are associated with different advantages and disadvantages for SCI therapy [10]. For example, embryonic stem cells (ESCs) exhibit high proliferative and pluripotent potential, but are associated with ethical concerns and the risk of immune rejection and teratoma formation [11]. Adult stem cells, including mesenchymal stem cells (MSCs) and oligodendrocyte precursor cells (OPCs), exhibit reduced immunogenicity and tumorigenicity. However, these cells also exhibit limited capacity for differentiation and integration [12, 13]. Schwann cells (SCs), olfactory ensheathing cells (OECs), NSCs, and umbilical cord blood derived cells (UCBDCs) are other cell types that have been tested for SCI therapy, with varying degrees of success and limitations [14–16]. In addition, hair follicle stem cells (HFSCs) and epidermal neural crest stem cells (EPI-NCSCs) are currently being investigated as potential cell-based therapies for SCI [17, 18] (Table 1).

**Table 1:** Cell type comparison

Cell type	Advantages	Disadvantages	Reference
ESCs	High proliferative and pluripotent potential; can differentiate into various types and region-specific NS/PCs	Ethical concerns; risk of immune rejection and teratoma formation	[11, 126–129]
MSCs	Low immunogenicity and tumorigenicity; easy to isolate and expand; secrete neurotrophic factors and anti-inflammatory cytokines; can differentiate into osteoblasts, chondrocytes, adipocytes, and myocytes	Low differentiation and integration capacity; limited survival and engraftment in vivo; variable quality and potency	[12, 130–135]
SCs	Can remyelinate host axons and enhance axonal regeneration; secrete neurotrophic factors and inhibit scar formation; can be derived from peripheral nerves or iPSCs	Limited sources and availability; risk of immune rejection and tumor formation for allogeneic SCs; may cause aberrant sprouting or synaptogenesis	[14, 136–141]
OECs	Can remyelinate host axons and enhance axonal regeneration; secrete neurotrophic factors and inhibit scar formation; can cross the glial scar and guide axonal growth; can be derived from olfactory mucosa or iPSCs	Limited sources and availability; risk of immune rejection and tumor formation for allogeneic OECs; may cause aberrant sprouting or synaptogenesis	[15, 142–147]
UCBDCs	Abundant and accessible sources; low immunogenicity and tumorigenicity; secrete neurotrophic factors and anti-inflammatory cytokines; can differentiate into neurons, glia, and endothelial cells	Low differentiation and integration capacity; limited survival and engraftment in vivo; variable quality and potency	[16, 148–155]
HFSCs	Low immunogenicity and tumorigenicity; easy to isolate and expand; can differentiate into epidermal cells and sebaceous gland cells; can be reprogrammed into iPSCs	Low differentiation and integration capacity; limited survival and engraftment in vivo; may cause hair overgrowth or cyst formation	[17, 156–162]
EPI-NCSCs	Low immunogenicity and tumorigenicity; easy to isolate and expand; can differentiate into neurons, glia, melanocytes, and smooth muscle cells; can be reprogrammed into iPSCs	Low differentiation and integration capacity; limited survival and engraftment in vivo; may cause pigmentation or tumor formation	[18, 163–167]

Several types of cells have been used for cell transplantation therapy in SCI. However, the choice of cell type for transplantation is crucial, as different cell types have different advantages and disadvantages for SCI therapy. This table summarizes the main features of some common cell types for SCI therapy, such as embryonic stem cells (ESCs), mesenchymal stem cells (MSCs), Schwann cells (SCs), olfactory ensheathing cells (OECs), umbilical cord blood derived cells (UCBDCs), hair follicle stem cells (HFSCs) and epidermal neural crest stem cells (EPI-NCSCs)

Of these various cell types, induced pluripotent stem cells (iPSCs) are currently regarded as highly promising for the treatment of SCI. iPSCs are generated by reprogramming somatic cells using specific transcription factors, including OCT4, SOX2, KLF4, and c-MYC (OSKM) [19]. Furthermore, iPSCs share key characteristics with ESCs, particularly their unlimited self-renewal capacity and potential for multi-lineage differentiation. Crucially, iPSCs circumvent the ethical concerns and immunological complications that are often associated with ESCs [20]. In addition, iPSCs can also be differentiated into various types and region-specific neural stem/progenitor cells (NS/PCs) which can then be tailored to the specific needs of SCI patients according to the site and extent of injury [4].

However, the use of iPSC-derived NS/PCs to treat SCI still faces many challenges and problems [21]. For example, there is a need to improve the generation efficiency and quality of iPSCs, to select the optimal differentiation protocol and culture conditions, and to prevent immune rejection and tumor formation after transplantation. One notable challenge is the epigenetic regulation and associated factors that could influence iPSC-derived NS/PCs in SCI treatment. Epigenetic inheritance refers to a genetic mechanism that regulates gene expression and function without altering DNA sequences; rather, this mechanism generates dynamic and reversible changes in DNA methylation, histone modifications, and chromatin structure [22, 23]. Epigenetics is known to play an important role in the generation, differentiation, and transplantation of iPSCs, and can influence the quality, safety, and efficacy of NS/PCs derived from iPSCs [24, 25]. Therefore, it is important to gain a comprehensive understanding of the epigenetic regulation and factors influencing iPSC-derived NS/PCs in the treatment of SCI at the molecular and functional levels if we are to optimize the preparation and transplantation protocols of iPSC-derived NS/PCs and improve the efficacy and safety of SCI therapy.

The aim of this article to provide a comprehensive overview of the epigenetic regulation and factors influencing iPSC-derived NS/PCs in SCI therapy, highlighting the current advances and challenges in this field, and identifying future directions and opportunities for improving the efficacy and safety of iPSC-based cell therapy for SCI. First, we introduce the epigenetic regulation of iPSC-derived NS/PCs in SCI therapy, including epigenetic reprogramming during the generation of iPSCs, epigenetic regulation during the differentiation of iPSCs, and the epigenetic adaptation of iPSC-derived NS/PCs in vivo after transplantation. Then, we introduce the factors affecting the epigenetic status of iPSC-derived NS/PCs in SCI therapy, including the impact of the epigenetic status of iPSC-derived NS/PCs on the efficacy and safety of SCI therapy, and the impact of other therapeutic tools (e.g., drugs, electrical stimulation, and scaffolds) on the epigenetic status of iPSC-derived NS/PCs in SCI therapy. We conclude by summarizing the main findings and implications of this article and idenfify future challenges and directions that need to be addressed and explored in this field.

### Epigenetic reprogramming during the generation of iPSCs

**Fig. 1:**
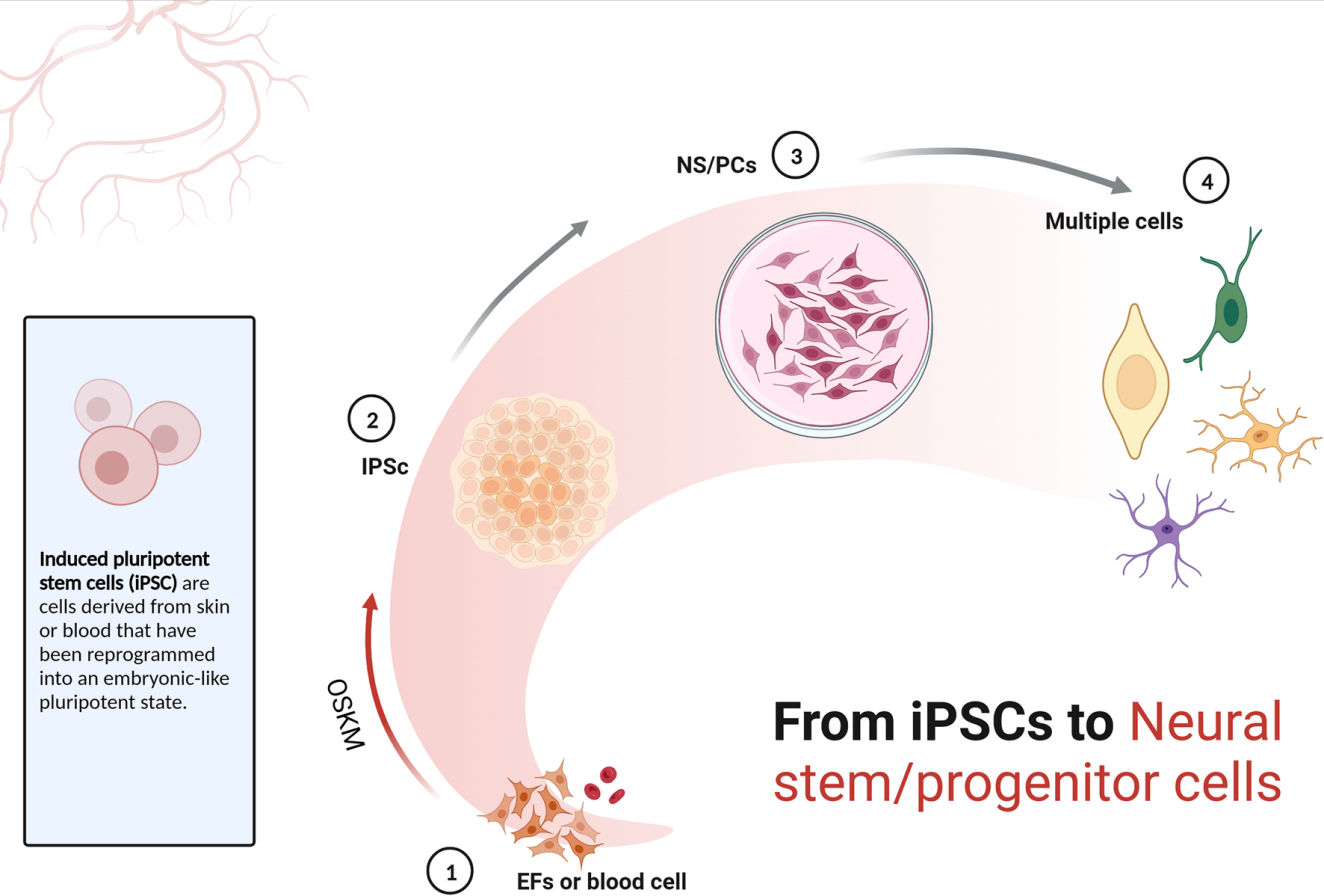
From IPSCs to neural stem/progenitor cells. This figure shows a schematic diagram of the process of differentiating induced pluripotent stem cells (iPSCs) derived from embryonic fibroblasts (EFs) or blood cells into neural stem/progenitor cells (NS/PCs) using different differentiation factors and culture conditions. The figure also shows that iPSCs and NS/PCs can further differentiate into various cell types, such as neurons, astrocytes and oligodendrocytes, during the differentiation process. This figure was made using BioRender, a web tool for creating scientific illustrations

**Fig. 2:**
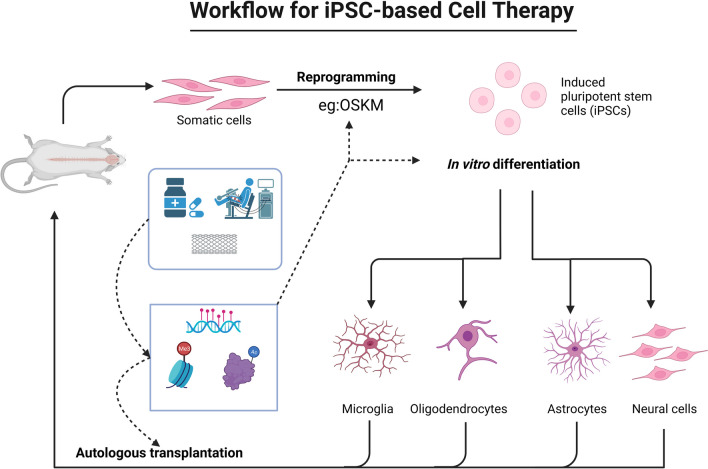
Workflow for iPSC-based cell therapy. This figure illustrates the steps and methods of using iPSCs to prepare NS/PCs and transplant them to treat SCI. And also show therapies (such as drugs, electrical stimulation, scaffolds, etc.) can modulate epigenetic regulation, which includes DNA methylation, histone modification and chromatin structure. This figure was made using BioRender, a web tool for creating scientific illustrations.

IPSCs are artificially created by introducing external factors such as OSKM into somatic cells, such as skin or blood cells [25–27]. In addition to standard transcription factors, various other elements play a role in regulating reprogramming, including growth factors, cytokines, and small molecules. These factors influence cell fate and function by affecting cellular metabolism, signaling pathways, and the structure of chromatin. The reprogramming process entails epigenetic modifications in the genome, including the resetting of DNA methylation, the alteration of histone modifications, and the restructuring of chromatin. These changes are crucial because they erase the original cellular identity and memory, activate genes related to pluripotency, and repress genes involved in differentiation [27, 28] (Figs. 1 and 2). However, epigenetic reprogramming does not reach completion during the generation of iPSCs, thus resulting in some epigenetic differences between iPSCs and ESCs. These epigenetic differences may cause heterogeneity in the pluripotency and differentiation capacity of iPSCs, as well as instability and tumorigenicity of iPSCs. Therefore, to improve the efficacy and safety of iPSCs in SCI therapy, it is necessary to optimize the methods we use to generate iPSCs, and to reduce the epigenetic differences between iPSCs and ESCs, and the epigenetic variation within iPSCs.

Different reprogramming methods, factors and conditions can cause epigenetic heterogeneity and instability in iPSCs, thus affecting their differentiation efficiency and quality when differentiated into NS/PCs. Therefore, selecting the most appropriate iPSC source, as based on the specific site of injury and severity, is crucial; these sources include skin cells, blood cells, and pancreatic beta cells. To improve iPSC generation in terms of both efficiency and quality, it is crucial to carefully select reprogramming factors, vectors, and optimize the conditions used for induction [29, 30]. Generally, a larger number or stronger reprogramming factors can improve the efficiency and quality of iPSC generation, although this practice can increase the risk of tumor formation or the formation of ectopic tissue. Safer or more efficient vectors can avoid insertional mutations in the genome or sustained expression, but reduce the efficiency and stability of iPSC generation. [30–33]. More suitable or regulated induction conditions can promote epigenetic reprogramming during the generation of iPSCs, but can also increase the heterogeneity or instability of iPSCs [33].

The epigenetic status of iPSCs reflects the completeness and quality of their reprogramming, as well as their potential for differentiation and direction into NS/PCs. Therefore, it is necessary to use comprehensive and accurate methods to detect and analyze the epigenetic status of iPSCs so that we can ensure their safety and efficacy for the treatment of SCI. Common evaluation methods include genome-wide methylation profiling, histone modification profiling and chromatin accessibility profiling; these methods can measure the level and pattern of DNA methylation, the type and location of histone modification, the chromatin structure and openness of iPSCs, respectively, and allow comparison with ESCs or somatic cells to determine whether iPSCs have a similar epigenetic status to ESCs [34, 35]. Common evaluation methods include genome-wide methylation profiling, histone modification profiling and chromatin accessibility profiling [36]. Genome-wide methylation profiling measures the DNA methylation levels and patterns of iPSCs and compares them with ESCs or somatic cells to determine whether iPSCs feature erased cell type-specific methylation markers and have acquired a methylation status similar to ESCs. Histone modification profiling identifies the type and location of histone modifications in iPSCs and compares them with ESCs or somatic cells to determine whether iPSCs have erased cell type-specific histone modification marks and acquired a histone modification status that is similar to ESCs. Chromatin accessibility profiling evaluates the chromatin structure and openness of iPSCs and compares them with ESCs or somatic cells to determine whether iPSCs feature an erased cell type-specific chromatin structure and have acquired chromatin accessibility that is similar to that of ESCs [25, 26, 36–38].

Epigenetic reprogramming is a crucial step for generating iPSCs from somatic cells, as this process can determine their quality, safety and pluripotency. However, epigenetic reprogramming is often incomplete or inefficient, thus leading to residual epigenetic memory or aberrant epigenetic marks in iPSCs. These effects may impair their differentiation potential and therapeutic application. Therefore, it is essential to understand the molecular mechanisms and regulatory factors that regulate epigenetic reprogramming in order to optimize the generation of iPSCs and enhance their performance in regenerative medicine.

In this review, we summarize and discuss the recent advances and discoveries in this field. First, different methods of iPSC generation can exert different impacts on the efficiency and quality of epigenetic reprogramming. Traditional methods for gene transduction, which involve the introduction of induction factors via viral vectors or plasmids, pose certain risks of genomic instability and carcinogenicity. To address these issues, innovative approaches such as chemical induction, protein transduction, and gene editing have been developed. These methods can induce somatic cell reprogramming by non-genetic or direct genomic approaches, thereby improving the quality and safety of iPSCs [27]. Second, the epigenetic memory and heterogeneity of iPSCs are important factors that can influence their differentiation potential and safety profile during application. Epigenetic memory refers to the retention of the characteristics of the original somatic cells at the epigenetic level by iPSCs; this memory may result in the limitation or preference of iPSCs in certain directions of differentiation. Epigenetic heterogeneity refers to the inter-individual or intra-individual variations of iPSCs at the epigenetic level and may cause inconsistency or unpredictability of iPSC functionality. Strategies such as optimizing cell sources, culture conditions, and gene modifications, have been suggested to alleviate these issues and aim to improve the epigenetic reprogramming and stability of iPSCs [23, 25, 39]. Epigenetic editing technologies for iPSCs, involving tools such as the CRISPR-Cas9 system or epigenetic editors, offer vast potential for the enhancement of iPSC quality and functionality. These technologies enable the selective modification of epigenetic marks in iPSCs, including methylation, histone modification, and chromatin structure. These technologies can effectively erase the epigenetic memory of iPSCs, increase the pluripotency and differentiation efficiency of iPSCs, or endow iPSCs with specific epigenetic features to facilitate the directional differentiation and functional expression of iPSCs [40]. For SCI therapy, it is critical that we select optimal cell sources, reprogramming methods and quality control criteria for the generation of iPSCs, as different cell types and tissues may have different epigenetic characteristics and reprogramming efficiency. Furthermore, the potential risks and benefits of epigenetic memory or heterogeneity in iPSCs for SCI treatment need to be carefully evaluated, as these factors may influence the differentiation direction, outcome and compatibility of iPSC-derived NS/PCs.

### Epigenetic regulation of iPSCs during differentiation

To apply for SCI treatment, iPSCs need to undergo specific differentiation protocols and culture conditions if they are to differentiate into NS/PCs with the potential for neural regeneration. This process involves dynamic and specific changes in the epigenetic status of iPSCs to guide cell fate determination and maintenance. The differentiation of iPSCs into NS/PCs involves numerous epigenetic changes, including methylation, histone modifications and chromatin structure [41, 42]. Methylation is the addition of methyl groups to cytosine residues on DNA by methyltransferases, which usually results in gene silencing. Histone modification involves the addition or removal of chemical groups on histone tails, such as acetyl, methyl, and phosphate to lysine or arginine residues. This process is mediated by various enzymes and influences both chromatin compaction and transcriptional activity. Chromatin structure is the complex three-dimensional organization of DNA and histones and other non-histone factors that determines DNA accessibility and functionality [43–45]. These epigenetic marks undergo dynamic and specific changes during the differentiation of iPSCs into NS/PCs to activate or repress differential gene expression, thus directing cell fate determination and maintenance [41, 45].

The differentiation of iPSCs into NS/PCs is influenced by epigenetic regulation by both differentiation factors and culture conditions [46]. Exogenous signaling molecules can induce iPSCs to differentiate into specific cell types [47, 48]. Endogenous environmental factors can influence the differentiation process of iPSCs. Furthermore, the epigenetic status of iPSCs during their differentiation into NS/PCs depends on differentiation factors and culture conditions, which can both influence the differentiation efficiency and quality of iPSCs [49–51]. For example, vitamin C is known to promote DNA demethylation and enhance the pluripotency and plasticity of iPSCs during their differentiation into NS/PCs [52]. Histone H3K4me3 and H3K27ac marks in the promoter and enhancer regions of genes related to neural development increase with retinoic acid (RA) and sonic hedgehog signaling pathway (SHH) [53]. The number of histone H3K27me3 marks in the promoter region of genes related to neural development are reduced significantly by ALK inhibitors and BMP inhibitors, thus influencing the differentiation of iPSCs into NS/PCs [54] Wnt3a and FGF8 reduce the levels of DNA methylation in the promoter region of these genes [55]. NGF (nerve growth factor) increases the open regions of chromatin regions (ATAC-seq) near these genes [56] Low oxygen concentration (5%) reduces the expression and activity of DNA methyltransferase DNMT1, which can reduce the DNA methylation level of these genes. High oxygen (20%) is known to upregulate the DNA methyltransferases DNMT3A and DNMT3B, subsequently increasing the DNA methylation level of these genes. High temperature (39 °C) is known to increase the levels of H3K9me3 and suppress the expression of genes related to development. Low temperature (32 °C) is known to reduce the levels of H3K9me3 and enhance the expression of genes related to neural development. Polyethylene glycol (PEG) is known to increase the open regions of chromatin (ATAC-seq) in the proximity or genes related to neural development, thus promoting the differentiation of iPSCs into NS/PCs. In contrast, collagen (COL) is known to reduce open regions of chromatin, thus inhibiting the differentiation of iPSCs into NS/PCs [18, 30].

The epigenetic regulation of iPSCs during their differentiation into NS/PCs is influenced by various differentiation factors and culture conditions, which can influence the methylation, histone modification and chromatin structure of iPSCs and their differentiation. These epigenetic changes can alter the gene expression and functionality of iPSCs and their derived NS/PCs, thus influencing their differentiation potential, direction and outcome. Thus, optimizing the differentiation factors and culture conditions to encourage the differentiation of iPSCs into NS/PCs is crucial if we are to improve the quality and efficiency of iPSC-derived NS/PCs for the treatment of SCI. However, there is still a lack of systematic and comprehensive studies relating to the specific mechanisms, optimal combinations and most suitable timing and doses of differentiation factors and culture conditions for the differentiation of iPSCs into NS/PCs. Furthermore, it remains unclear as to how combinations of different growth factors, including vitamin C, RA, SHH, ALK inhibitors, BMP inhibitors, Wnt3a, FGF8, and NGF, can influence the DNA methylation, histone modifications, and chromatin structure in iPSCs and NS/PCs during differentiation. In addition, the role of these epigenetic marks in regulating gene expression and function related to pluripotency and neurodevelopment requires further investigation. The impact of varying culture conditions, including oxygen concentration, temperature, and substrate type, on the epigenetic status and differentiation outcomes of iPSCs and NS/PCs is not yet fully understood. Furthermore, it remains to be determined if there are species-specific or individual-specific variations in the epigenetic responses of iPSCs and NS/PCs to these conditions. Thus, further experimental and clinical research is essential if we are to identify the most effective epigenetic regulation strategies for differentiating iPSCs into NS/PCs for the treatment of SCI. This research should include the development of standardized and personalized protocols, the application of epigenetic editing tools, and integrating these approaches with other therapeutic modalities.

The epigenetic regulation of iPSCs during their differentiation into NS/PCs can be evaluated by investigating and analyzing the genomes of iPSCs and NS/PCs using the same methods as those used to generate iPSCs, including genome-wide methylation profiling, histone modification profiling and chromatin accessibility profiling [54]. These methods can reveal changes and differences in the epigenetic status of iPSCs during their differentiation into NS/PCs and their association with gene expression and cellular functionality. For example, one study showed that DNA methylation levels decreased significantly during the differentiation of iPSCs into NS/PCs, especially in the promoter regions of genes related to pluripotency and neurodevelopment; furthermore, the expression of these genes increased accordingly, thus facilitating the neural differentiation of iPSCs [53]. Another study showed that histone modifications changed markedly in both type and location during the differentiation of iPSCs into NS/PCs, especially H3K4me3 and H3K27me3, two histone modifications with opposing functions, which exhibited mutually exclusive or co-occurring patterns in the promoter regions of genes associated with pluripotency and neurodevelopment, thus modulating the neural differentiation of iPSCs [57]. Another study showed that chromatin structure and accessibility changed substantially during the differentiation of iPSCs into NS/PCs, especially in the promoter regions of genes related to pluripotency and neurodevelopment; furthermore, these genes exhibited a more open and active chromatin state, thus enhancing the neural differentiation of iPSCs [58].

The field of iPSCs and epigenetics is rapidly evolving and offers many opportunities for advancing our understanding of cellular reprogramming and differentiation. There are several future directions that could be explored. For example, we need to determine how different reprogramming methods can affect the epigenetic landscape and differentiation potential of iPSCs. For example, chemical reprogramming has been shown to generate iPSCs with less epigenetic memory and more differentiation potential than OSKM reprogramming [59]. We also need to determine the mechanisms and consequences of epigenetic memory in iPSCs derived from different cell types and tissues. For example, iPSCs derived from human pancreatic islet β cells (BiPSCs) have been found to exhibit enhanced and reproducible differentiation into insulin-producing cells [60]. Furthermore, there is a need to identify which somatic driver mutations are recurrent in iPSCs and how they might affect their functionality. For example, BCL6 Interacting Corepressor (BCOR) mutations have been reported frequently and can impair the differentiation capacity of iPSCs [61]. These research directions are important if we are to enhance the quality and safety of iPSC-based applications in regenerative medicine and disease modeling. To address these questions, it will be necessary to apply a combination of genomic, epigenomic and transcriptomic analyses, as well as functional assays. Moreover, more standardized protocols for iPSC generation, maintenance and differentiation would be beneficial if we are to reduce the variability and heterogeneity among iPSC lines.

### Epigenetic adaptation of iPSC-derived NS/PCs *in viv*o after transplantation

Following transplantation into SCI patients or animal models, iPSC-derived NS/PCs face a different neural tissue environment in vivo than in in vitro culture conditions. This may lead to adaptive changes in the transplanted cells at the epigenetic level, which regulate their differentiation, migration, survival and interaction with host tissues [62–64]. Epigenetic adaptations may vary among species, thus affecting the efficacy and safety of transplanted cells for the treatment of SCI [65, 66]. The differentiation direction and efficiency of iPSC-derived NS/PCs in SCI therapy are important factors that can influence their therapeutic effects. Generally, iPSC-derived NS/PCs differentiate into neurons and glial cells after transplantation, thus promoting neural repair in the area of the SCI lesion. However, the differentiation direction and efficiency of iPSC-derived NS/PCs are influenced by various factors, particularly epigenetic status. Research has shown that epigenetic status can influence the differentiation potential and fate of iPSC-derived NS/PCs, as well as their ability to adapt to the post-transplantation environment. Therefore, controlling and regulating the epigenetic status of iPSC-derived NS/PCs is of great significance if we are to improve their differentiation direction and efficiency in SCI therapy.

In a mouse model, iPSCs-derived NS/PCs exhibited significant changes in DNA methylation levels after transplantation, predominantly in the promoter regions of genes related to neurodevelopment. This may be related to the differentiation ability of transplanted cells. Moreover, the histone modifications of transplanted cells also underwent alterations, predominantly involving H3K4me3, H3K27me3 and H3K9me3. This may be related to the migration and survival of transplanted cells. In a monkey model, iPSCs-derived NS/PCs also exhibited changes in DNA methylation levels following transplantation, but unlike the mouse model, these changes were mainly concentrated in the promoter regions of genes related to neurological function. This may be related to the ability of transplanted cells to interact with the host tissue. In addition, histone modifications in the transplanted cells also underwent changes; however, unlike the mouse model, these changes predominantly involved H3K36me3 and H4K20me3. This may be related to the viability and safety of transplanted cells. Few studies have investigated the epigenetic adaptations of iPSC-derived NS/PCs in vivo following transplantation in human models, and only a handful of clinical trials have investigated this issue [20, 64, 67–70]. For example, a clinical trial in Japan transplanted iPSC-derived NS/PCs into patients with subacute complete SCI to enhance neural regeneration and functional recovery. This trial began in 2020 and ended in 2023 [9]. Another clinical trial in the USA transplanted iPSC-derived intermediate stromal neurons (MSNs) into patients with Huntington’s disease (HD) to replace damaged striatal neurons. This trial started in 2019 and ended in 2023 [71]. A third clinical trial in China is transplanting iPSC-derived motor neuron precursor cells (MNPs) into patients with spinal muscular atrophy (SMA) to increase motor neuron number and function. The trial started in 2017 and has completed Phase I and Phase II with positive results in relation to safety and efficacy [72].

The epigenetic status of iPSCs-derived NS/PCs in vivo after transplantation depends not only on their own characteristics but also on various external factors [73, 74]. These factors include the host neural tissue environment, the site and extent of injury, and the transplantation timing and dose [75–77]. For example, the transplantation timing affects the differentiation direction and outcome of iPSC-derived NS/PCs in vivo. Transplantation is most effective in the subacute phase (2–4 weeks after injury) and less effective in the acute phase (1–2 weeks after injury) or chronic phase (more than 6 months after injury). Similarly, the transplantation dose affects the survival and migration ability of iPSC-derived NS/PCs in vivo. A low dose (1 million) is known to be better than a high dose (5 million) for human patients, probably because of the hypoxic and inflammatory response at the transplantation site induced by the high dose [9, 74, 78]. The transplantation route refers to the manner in which iPSC-derived NS/PCs are injected into the injured spinal cord, and includes intraspinal transplantation and extraspinal transplantation. Intraspinal transplantation is the injection of cells directly into the injured spinal cord; this can place the cells closer to the injury site, but may also cause further damage or bleeding [9]. Extraspinal transplantation involves the injection of cells into the tissues surrounding the injured spinal cord, including the subdural space, epidural space or perispinal fat; this method can avoid direct damage to the spinal cord, but may also reduce the migration and differentiation ability of the cell [69]. Few studies have investigated the impact of transplantation route on the in vivo epigenetic adaptation and therapeutic effects of transplanted cells; consequently, there is a need for further research in this area.

To investigate the in vivo epigenetic adaptation of iPSC-derived NS/PCs post-transplantation, it is necessary to utilize comprehensive evaluation methods similar to those used during the generation and differentiation of iPSCs. These include the analysis of genome-wide methylation profiles, histone modification profiles, and chromatin accessibility profiles [79–81]. These methods allow the comprehensive and precise detection and analysis of the epigenetic status of transplanted cells in vivo, thus revealing the interaction mechanisms and signaling pathways between transplanted cells and host neural tissue [82]. For example, Goldenson et al. used bisulfite sequencing and chromatin immunoprecipitation sequencing (ChIP-seq) to analyze the genomic methylation and histone modification levels of iPSC-derived NK cells in vivo. These authors identified several epigenetic marks associated with NK cell-specific genes and functions [83]. Similarly, Efrat used assay for transposase-accessible chromatin using sequencing (ATAC-seq) methodology to analyze chromatin accessibility changes in BiPSCs after differentiation into islet cells. These authors identified a number of differentially open chromatin regions (DOCs) associated with β-cell-specific genes [84].

In addition to using these methods to evaluate the epigenetic adaptation of iPSCs-derived NS/PCs in vivo after transplantation, other criteria and metrics are also required to investigate the efficacy and safety of transplanted cells in SCI therapy by measuring their ability to differentiate, migrate, survive and interact with host neural tissue in vivo [85, 86]. Various criteria and techniques have been employed to investigate transplanted cells and their integration with host neural tissue. These methods include labeling cells with specific antibodies or fluorescent proteins, or the utilization of immunofluorescence or immunohistochemistry to observe cell distribution, survival, differentiation, and connections with host axons or blood vessels. In addition, electrophysiological or behavioral methods can be used to investigate the recovery of neurological function. Techniques such as PCR or western blotting can also be used to analyze in vivo gene expression and signaling pathway activity in transplanted cells [80, 87, 88].

The epigenetic adaptation of iPSC-derived NS/PCs in vivo after transplantation is a complex and dynamic process that influences the functional integration and neurological recovery of transplanted cells in SCI therapy. To optimize the therapeutic outcomes of SCI treatment, it is crucial to understand how the epigenetic status of transplanted cells influences their function and fate, and how these outcomes can be modulated. Some of the future directions and challenges in this field include: (1) developing more reliable and sensitive methods to detect and evaluate the epigenetic status of transplanted cells in vivo; (2) identifying the key epigenetic marks and pathways that regulate the differentiation, migration, survival and interaction of transplanted cells with host neural tissue; (3) exploring the interaction and synergy between the epigenetic status of transplanted cells and other therapeutic tools (e.g., drugs, electrical stimulation, and scaffolds); (4) establishing standardized and personalized protocols for the generation, differentiation and transplantation of iPSCs based on the epigenetic characteristics of different cell sources and patients; and (5) conducting further preclinical and clinical trials to investigate the safety and efficacy of iPSC-derived NS/PCs transplantation for SCI treatment in different models and settings.

### Impact of epigenetic status of NS/PCs derived from iPSCs on the efficacy and safety of SCI treatment

Evaluating the epigenetic status of transplanted cells in SCI therapy is an important and challenging task, as it can provide valuable information for the assessment of cell identity, quality, stability and compatibility, and could be used to predict therapeutic outcomes and potential complications. The identity of transplanted cells, defined by their specific type and characteristics, such as being region-specific, subtype-specific, or interneuron-specific NS/PCs, is crucial in determining their differentiation capabilities and ability to functionally integrate with the host neural tissue Cell quality refers to the purity and consistency of transplanted cells, which can affect their survival and migration ability and their risk of tumorigenesis or immunogenicity. Cell stability refers to the maintenance and adaptation of transplanted cells with regards to their in vivo epigenetic status; these factors can influence their long-term functionality and fate. Cell compatibility refers to the interaction and synergy of transplanted cells with host neural tissue and other therapeutic tools, which can modulate their epigenetic status and therapeutic efficacy. Therapeutic outcomes refer to the degree and extent of neurological recovery and the improvement of SCI patients or animal models after transplantation; the effect of these factors depend on the epigenetic status and functionality of transplanted cells. Potential complications from transplantation encompass various adverse effects and risks, including infection, inflammation, scar and tumor formation, or immune rejection. These complications may be linked to the epigenetic status and quality of the transplanted cells. The efficacy and safety of iPSC-derived NS/PCs in SCI treatment are influenced by their epigenetic characteristics, such as DNA methylation, histone modifications, and chromatin structure. These cells must navigate the complex microenvironment at the injury site and effectively interact with host neural tissues to aid in neurological recovery [89–91] (Figs. 1 and 2).

Several studies have shown that the epigenetic status of iPSC-derived NS/PCs can influence their ability to differentiate, migrate, survive and interact with host neural tissue during SCI treatment [92–94]. Methylation, a common DNA epigenetic modification, is known to regulates gene expression. Abnormal levels of methylation may cause uncontrolled or incorrect differentiation of transplanted cells, thus impairing their ability to replace damaged neural tissues or reconstruct neural circuits. Histone modifications, a common epigenetic modification of the chromatin, can regulate the structure and function of chromatin. Abnormal levels or patterns of histone modifications may reduce the migration or survival of transplanted cells, thus limiting their ability to reach or adapt to the site of injury. Chromatin structure, a high-level epigenetic hierarchy, is known to regulate genomic accessibility and stability. Furthermore, abnormal chromatin structure may impair the interaction of transplanted cells with host neural tissue, thus hindering their ability to promote host axon remyelination or inhibit inflammation and scar formation [94–97].

The epigenetic status of iPSC-derived NS/PCs can also influence safety issues and neurological recovery during SCI therapy [98–101]. For example, undifferentiated iPSCs or other heterogeneous cells with unlimited proliferative capacity and multidirectional differentiation potential may cause tumor formation or the formation of ectopic tissue after transplantation. Gene expression in transplanted cells may influence the abnormal levels or patterns of methylation or histone modification, which may cause immune mismatch with the host tissue and result in immune rejection or tolerance. An abnormal chromatin structure may compromise the genomic stability or integrity of transplanted cells, which may increase the risk of infection or loss of function. Moreover, abnormal levels or patterns of methylation or histone modifications may alter the levels of gene expression in transplanted cells, thus affecting the synthesis or release of nerve growth factors or neurotransmitters, thereby influencing nerve signaling and neuroplasticity. An abnormal chromatin structure may also impair the adaptation or stress response of transplanted cells to the microenvironment at the site of injury, thus affecting neuroprotection and neurorepair [77, 86, 102, 103].

### Effect of other treatments (e.g., drugs, electrical stimulation, and stents) on the epigenetic status of iPSC-derived NS/PCs in SCI treatment

The epigenetic status of iPSC-derived NS/PCs can also be influenced by other therapeutic tools (e.g., drugs, electrical stimulation, and scaffolds) in SCI treatment, which may alter the differentiation direction, activity and interaction mechanisms of transplanted cells, thus producing synergistic effects or optimized strategies [4, 104–106] (Figs. 1 and 2).

However, our current knowledge and understanding of the effects of other treatments on the epigenetic status of iPSC-derived NS/PCs in SCI treatment remains very limited, as most previous studies have focused on the effect of single or a limited number of treatments, and have used different methods and models to evaluate the epigenetic status and functionality of transplanted cells. Therefore, more systematic and comprehensive studies are now needed to identify the optimal combinations and timing of co-treatments, to elucidate the synergistic or antagonistic effects of co-treatments on the epigenetic status and functionality of transplanted cells, and to develop novel epigenetic modulators or sensors that can enhance or monitor the efficacy and safety of transplanted cells in SCI therapy.

Drugs are a common therapeutic tool that can modulate signaling pathways, transcription factors and enzyme activities to regulate the epigenetic status of iPSC-derived NS/PCs. Previous research demonstrated that γ-secretase inhibitors could reduce the methylation level of iPSC-derived NS/PCs and enhance their differentiation into neurons and oligodendrocytes, thus improving neurological recovery. In addition, RA has also been shown to induce histone acetylation and demethylation to promote the neural differentiation of iPSC-derived NS/PCs [106–109] (Fig. 2). Other research has shown that methylprednisolone, a drug that is frequently administered during SCI treatments, may hinder the differentiation of iPSC-derived NS/PCs into oligodendrocytes. This effect is attributed to an increased DNA methylation level in oligodendrocyte-specific genes, such as MBP and PLP [110]. Another study found that ganglioside GM1, a drug that can promote the metabolism and repair of nerve cells, could activate the histone acetylation level of neuron-specific genes, such as β-III-tubulin and MAP2, to promote the differentiation of iPSCs-derived NS/PCs into neurons. Other drugs, such as scopolamine, cobalamin, and mannitol, might also influence the epigenetic status of iPSC-derived NS/PCs. However, their specific mechanisms and impacts need to be investigated further [111].

Electrical stimulation is a physical therapy that can stimulate the activity of neurons and axons to influence the epigenetic status of iPSC-derived NS/PCs. One study found that the use of synthetic receptor techniques (DREADDs) to stimulate the activity of human iPSC-derived NS/PCs transplanted into a mouse SCI model increased synaptic activity between the transplanted cells and the host neural tissue, thus resulting in improved motor functionality. Other research demonstrated that AC electric fields can alter chromatin structure and gene expression to promote the neural differentiation of iPSC-derived NS/PCs [112–117].

Scaffolds are a bioengineering tool that can provide physical support, release growth factors and regulate intercellular interactions to influence the epigenetic status of iPSC-derived NS/PCs. For example, one study found that co-transplanting human iPSC-derived NS/PCs with poly lactic acid-hydroxyacetic acid copolymer (PLGA) scaffolds into a rat SCI model increased the survival and differentiation of transplanted cells at the site of injury, thereby promoting neurological recovery. Another study found that nanofiber scaffolds could mimic the microenvironment of neural tissue to promote the migration and neural differentiation of iPSC-derived NS/PCs [95, 99, 103, 118, 119].

Collectively, these findings suggest that other therapeutic tools can have an important impact on the epigenetic status of iPSC-derived NS/PCs in SCI treatment, thus altering the differentiation direction, activity and interaction mechanisms of transplanted cells to produce synergistic effects or optimization strategies. However, there is a lack of systematic studies and evidence relating to the specific mechanisms of action, optimal combinations, and the most suitable timing and dose of transplantation for these therapeutic tools. Therefore, further experimental and clinical trials are now needed to investigate the effects and optimization strategies of other therapeutic tools (e.g., drugs, electrical stimulation, and scaffolds) on the epigenetic status of iPSC-derived NS/PCs to improve the efficacy and safety of transplanted cells for SCI treatment.

To investigate the effects of other therapeutic tools (e.g., drugs, electrical stimulation, and scaffolds) on the epigenetic status of iPSC-derived NS/PCs in SCI treatment, it is necessary to develop validated methods and criteria. However, unlike the epigenetic methods used during the generation and differentiation of iPSCs, the epigenetic methods used in vivo after transplantation need to account for the effects of the neural tissue environment in the host, the site and extent of injury, along with the transplantation timing and dose on the epigenetic status of transplanted cells, as well as the effects of the epigenetic status of transplanted cells on the recovery of neurological function. Currently, there are several methods for epigenetic detection and evaluation in vivo after transplantation. Genome-wide methylation profiling detects the methylation levels of all CpG sites on the genome and reflects the methylation status and pattern of iPSC-derived NS/PCs. Techniques such as bisulfite sequencing (BS-seq), reduced representation bisulfite sequencing (RRBS-seq), and methylated DNA immunoprecipitation sequencing (MeDIP-seq) can also be used for this purpose [120, 121]. This method can investigate the methylation reprogramming or adaptation of transplanted cells in vivo and their immune rejection response from the host neural tissue. Histone modification profiling can determine the levels of different types and locations of modifications on histones, thus reflecting the status and pattern of histone modifications in iPSC-derived NS/PCs. This method can involve numerous approache, including chromatin immunoprecipitation sequencing (ChIP-seq) or histone modification analysis via mass spectrometry (HMA-MS) [122, 123]. This method can also determine the histone modification reprogramming or adaptation of transplanted cells in vivo and their epigenetic interactions with neural tissue in the host. Chromatin accessibility profiling can reveal the accessibility levels of different regions on the chromatin, thus reflecting the state of chromatin structure and pattern of iPSC-derived NS/PCs. TO implement this technique, it is possible to utilize techniques such as DNase I hypersensitive site sequencing (DNase-seq), ATAC-seq, formaldehyde-assisted isolation of regulatory elements sequencing (FAIRE-seq), or the formaldehyde-assisted isolation of iPSCs [124, 125]. This technique is capable of analyzing the reprogramming of chromatin structure or the adaptation of transplanted cells in vivo, as well as their transcriptional regulatory interactions with neural tissue in the host. Although these methods can effectively elucidate the epigenetic status of iPSC-derived NS/PCs at various levels, these techniques are constrained by challenges such as the need for large sample sizes, complex procedures, and intricate data analysis. Consequently, there is a significant need to develop simpler, faster, more precise, and sensitive epigenetic techniques for broader application in both experimental and clinical contexts.

## Conclusion

In this article, we reviewed how epigenetic regulation and various factors can influence iPSC-derived NS/PCs during SCI therapy at the molecular and functional levels. We describe how the epigenetic reprogramming, regulation and adaptation of iPSCs during their generation, differentiation and transplantation can influence the differentiation, migration, survival and interaction of transplanted cells with neural tissue in the host, thus influencing the efficacy and safety of SCI therapy. We also described how other therapies (e.g., drugs, electrical stimulation, and scaffolds) can modulate and synergize with the epigenetic status of transplanted cells to enhance the efficiency and efficacy of neurological recovery.

We propose that the modification of iPSCs to render them more suitable for cell therapy is a promising direction for future research and development. There are several possible methods with which to generate such modifications, as follows: (1) using gene editing techniques to correct disease-causing mutations or improve desirable properties of iPSC-derived NS/PCs; (2) using chemical reprogramming methods to generate iPSCs with less epigenetic memory and higher differentiation potential; (3) using fit-for-all iPSCs with reduced immunogenicity by engineering the expression of HLA or immune checkpoint molecules; and (4) the use of humanized mice with reconstituted NK cells to evaluate the immune response and safety of iPSC-derived NS/PCs. These solutions may help us to overcome some of the current challenges and limitations of iPSC-based cell therapy for SCI treatment.

## Data Availability

Not applicable.

## Abbreviations

SCI: Spinal cord injury
NS/PCs: Neural stem/progenitor cells
iPSCs: Induced pluripotent stem cells
NSC: Neural stem cell
ESCs: Embryonic stem cells
MSCs: Mesenchymal stem cells
OPCs: Oligodendrocyte precursor cells
UCBDCs: Umbilical cord blood derived cells
HFSCs: Hair follicle stem cells
EPI-NCSCs: Epidermal neural crest stem cells
NGF: Nerve growth factor
BiPSCs: Human pancreatic islet β cells
BCOR: BCL6 Interacting Corepressor
HD: Huntington’s disease
MNPs: Motor neuron precursor cells
SMA: Spinal muscular atrophy
ChIP-seq: Chromatin immunoprecipitation sequencing
ATAC-seq: Assay for transposase-accessible chromatin using sequencing
DOCs: Differentially open chromatin regions
BS-seq: Bisulfite sequencing
RRBS-seq: Reduced representation bisulfite sequencing
MeDIP-seq: Methylated DNA immunoprecipitation sequencing
ChIP-seq: Chromatin immunoprecipitation sequencing
HMA-MS: Histone modification analysis via mass spectrometry
FAIRE-seq: Formaldehyde-assisted isolation of regulatory elements sequencing
